# Purine nucleoside phosphorylase deficiency in two unrelated Saudi patients

**DOI:** 10.4103/0256-4947.55320

**Published:** 2009

**Authors:** Abdullah Alangari, Abdullah Al-Harbi, Abdulaziz Al-Ghonaium, Ines Santisteban, Michael Hershfield

**Affiliations:** a Department of Pediatrics, King Saud University, Riyadh, Saudi Arabia; b Department of Pediatrics, Al-Hada Military Hospital, Taif, Saudi Arabia; c Department of Pediatrics, King Faisal Specialist Hospital, Riyadh, Saudi Arabia; d Department of Medicine, Duke University Medical Center, Durham, NC, USA

## Abstract

Purine nucleoside phosphorylase (PNP) deficiency is a rare autosomal recessive metabolic disorder that results in combined immunodeficiency, neurologic dysfunction and autoimmunity. PNP deficiency has never been reported from Saudi Arabia or in patients with an Arabic ethnic background. We report on two Saudi girls with PNP deficiency. Both showed severe lymphopenia and neurological involvement. Sequencing of the PNP gene of one girl revealed a novel missense mutation Pro146>Leu in exon 4 due to a change in the codon from CCT>CTT. Expression of PNP (146L) cDNA in *E coli* indicated that the mutation greatly reduced, but did not completely eliminate PNP activity.

Purine nucleoside phosphorylase (PNP) deficiency (OMIM 164050) is a rare autosomal recessive disorder of the purine metabolic pathway that results in combined immunodeficiency with a profound T-cell defect and variable B-cell dysfunction. It is also associated with neurologic dysfunction in about two thirds of patients and autoimmunity in about one third.^1^–^3^ PNP deficiency was first described by Giblett et al in 1975 shortly after their discovery of a deficiency of another enzyme of the purine metabolic pathway, adenosine deaminase (ADA).^4^ PNP catalyzes the reversible degradation of inosine and deoxyinosine to hypoxanthine and guanosine, and deoxyguanosine to guanine. When PNP activity is absent or greatly diminished, deoxyguanosine triphosphate is thought to accumulate in the mitochondria, which inhibits ribonucleotide reductase and mitochondrial DNA repair. This leads to increased sensitivity of T lymphocytes to DNA damage and apoptosis during thymus selection. PNP is encoded by six exons spanning 7.5 kb of chromosome 14q13. Approximately 17 disease-causing mutations in the PNP gene have been reported, and the somewhat variable course of the disease associated with PNP deficiency may be related to this genetic heterogeneity.^5^

Less than 50 patients with PNP deficiency have been reported in the literature. To our knowledge, PNP deficiency has never been reported in patients from Saudi Arabia or in patients with an Arabic ethnic background. We report clinical and immunologic findings in two unrelated Saudi girls with PNP deficiency. One of these patients was found to be homozygous for a novel PNP gene mutation.

## CASE 1

A 2-year-old Saudi girl was referred to King Khalid University Hospital with history of recurrent chest infections. She had three chest infections. The first at 11 months of age that was treated with oral antibiotics, while the second and third required prolonged courses of intravenous antibiotics. She also had chronic diarrhea, watery, 3 to 4 times per day, and persistent since she was 9 months of age. There was a history of intermittent anemia and leukopenia; she had received a blood transfusion 3 weeks prior to presentation. The patient's parents were first cousins. She has two healthy brothers who are 4 years and 8 months of age. One cousin died at 1 year of age after extensive chicken pox infection.

On examination, the patient appeared poorly nourished and growth parameters were all below the third percentile. A BCG scar was absent (the patient received BCG vaccine on day one of life). She had oral thrush and pus draining from the right ear. Chest examination revealed bilateral coarse crackles. CNS exam revealed a global developmental delay corresponding to about 9 months of age and universally decreased muscle tone.

Investigations showed WBCs of 1300/mm^3^, an absolute neutrophil count of 510 /mm^3^, absolute lymphocyte count 420 /mm^3^, hemoglobin of 11.3 g/dL, and platelets of 506×103/mm^3^. Bone marrow aspiration revealed a hypercellular active marrow with no dysplasia. Immunoglobulin levels were all elevated: IgG 1877 mg/dL, IgM 366 mg/dL, and IgA 297 mg/dL. Lymphocyte subset enumeration and proliferation responses are shown in Table 1. Uric acid was 62.6 μmol/L (normal range 130-320 μmol/L). PNP enzyme activity was measured by a radiochemical method,^6^ as adapted for measuring activity in eluates of dried blood spots on filter paper^7^ and it was 324 nmol/L (normal 1336±441). The low level of PNP activity detected, at least partly, reflected the recent blood transfusion that the patient had received because of her anemia.

**Table 1:** Immunological investigation data for the two patients.

		Patient 1	Patient 2	Normal range
Lymphocytes Subsets enumeration /mm^3^	-CD 3	385	314	1400-8000
	-CD 4	106	167	900-5500
	-CD 8	263	18	400-2300
	-CD 19	311	58	600-3100
	-CD 16/56	147	231	100-1400

Lymphocyte proliferation responses	-PHA (cpm)	2343	14247	90553-117987
	-Con A (cpm)	1443	10350	78412-105150
	-PWM (cpm)	2934	8705	45008-53536

Enzyme activity	PNP nmol/hr/mg	324	0	1336 (±441)

cpm: counts per minute.

## CASE 2

The second patient was referred to King Faisal Specialist Hospital at 2 years of age because of recurrent chest infections that required multiple hospital admissions for treatment with intravenous antibiotics. Her most recent chest infection was complicated by empyema. There was no history of chronic diarrhea. Her parents were first cousins. She has two brothers and one sister who are alive and well. Another sister died at 3 years of age from extensive chicken pox infection.

On examination, all her growth parameters were below the third percentile. She had delayed motor milestones. Speech was appropriate for age. The tonsils were absent. The remaining systemic examination was normal. Investigations showed WBCs of 4270/mm^3^, an absolute neutrophil count of 2540/mm^3^, absolute lymphocyte count 618/mm^3^, hemoglobin of 9.7 g/dL, and platelets of 643×103/mm^3^. Immunoglobulin levels showed IgG 1000 mg/dL (N), IgM 20 mg/dL (low), and IgA 14 mg/dL (normal). Antibody response titer to tetanus was 13.48 IU/mL (protective level>0.17 IU/mL). Lymphocyte subset enumeration and proliferation responses are shown in Table 1. Erythrocyte PNP enzyme activity was undetectable. Unfortunately, material for genotype analysis was not available at the time of diagnosis and the patient was lost to follow-up.

Both patients were vaccinated with BCG, OPV and MMR among other first year killed vaccines (DTP, HIB and Hep B) with no complications.

### Mutation analysis

Genomic DNA was prepared from blood samples of patient 1 by standard methods. The exons and intronexon boundaries of the PNP gene were amplified in 6 separate segments spanning exon 1, exon 2, exons 3-5, exon 6 and the promoter region. The resulting PCR fragments were sequenced on an ABI 3730 DNA Analyzer (Applied Biosystems, USA).

The primers used for the polymerase chain reactions were:

PNPex1:

5'(+) TCAGTTCAGCATAGCGGAG

5'(−) TCCACTGTCCCTAACACAC

PNPex2:

5'(+) TGCCTCAGTATACCTGCCAG

5'(−) GTCTTCAGTGAAATGCTAGGTC

PNPex3-5:

5'(+) AGCAGAGCGAGTAACTCAC

5'(−) TTCCTACTCCTTCCCCTTCC

PNPex6: 5'(+) TACAGGTGTGAACCACTGC

5'(−) AAGAAAGTGGGAAAGGTGAAG

PNPprom:

5'(+) CGATGAGCCTGCTCCGGTGT

5'(−) TCTAGGACCCCGGAATTCTT

### Expression analysis

PNP 146L cDNA was generated using the wild type PNP (146P) cDNA in the pZ vector as template and the Quick Change Mutagenesis kit from Stratagene.

The primers used to introduce the mutation were:

PNP146L:

5'(+) TTCAGTGGTCAGAACCth T TCTCAGAGGGCCCAAT

5'(−) ATTGGGCCCTCTGAGAAGGTTCTGACCACTGAA

The wild type (146P) and mutant (146L) PNP cDNAs were expressed in *E coli* SΦ3834 essentially as described for human adenosine deaminase.^8^ Human PNP activity in extracts of transformants was analyzed histochemically after electrophoresis of bacterial lysates on cellulose acetate strips as described.^6^ In situ staining was also performed as described for adenosine deaminase, except that inosine was used as the substrate and exogenous PNP was omitted.

**Figure 1:**
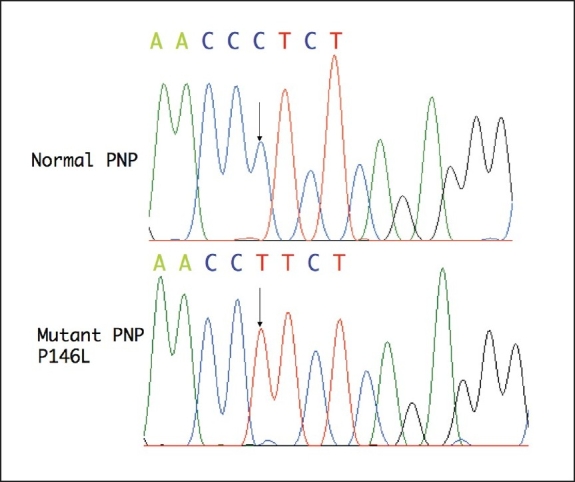
Sequencing of PNP gene from case 1 revealed a novel missense mution Pro146>Leu in exon 4 due to a change in the codon from CCT>CTT.

**Figure 2:**
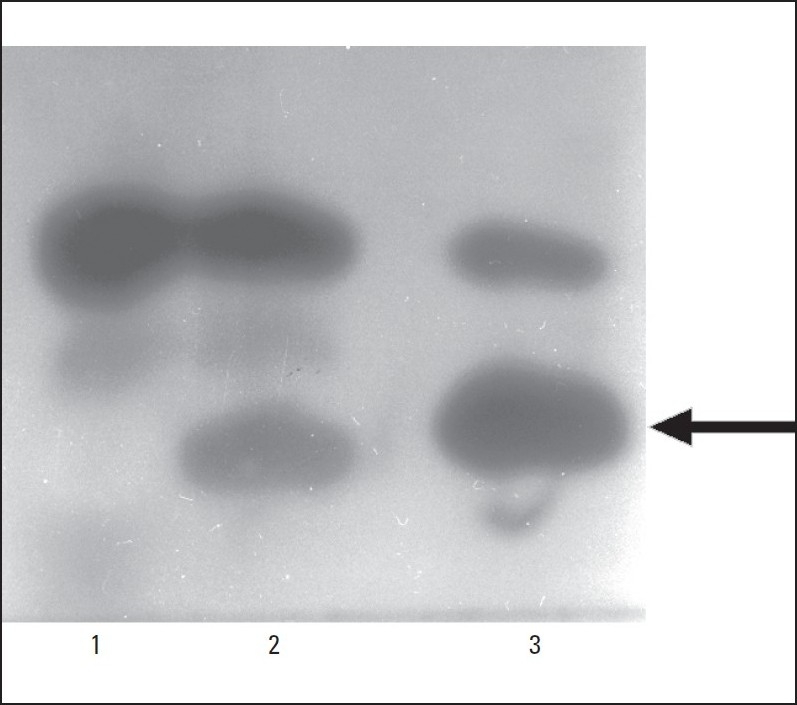
Histochemical in situ assay for PNP activity of recombinant wild type and P146L mutant human PNP, following expression in *E coli*. Lanes contained approximately equal amounts of extract protein. Lane 1, untransformed *E coli* SΦ3834 (control). Lane 2, *E coli* Sø3834 transformed with 146L (mutant) PNP. Lane 3, *E coli* Sø3834 transformed with 146P (wild type) PNP. The upper staining band is due to *E coli* PNP. The arrow indicates the position of human PNP.

Sequencing of the amplified exons and intron-exon boundaries of the PNP gene from genomic DNA from patient 1 revealed a novel missense mutation Pro146>Leu in exon 4 due to a change in the codon from CCT>CTT (Figure 1). Expression in *E coli* of human PNP cDNA carrying either the wild type PNP sequence or the P146L mutation revealed that the mutant protein had some residual PNP activity (Figure 2). The method used to assess the activity of the mutant enzyme is semiquantitative, but these results suggest that the mutation probably does not completely eliminate PNP activity.

## DISCUSSION

PNP deficiency is one of the least common primary immunodeficiency diseases. Seventeen different mutations in 23 unrelated individuals have been described so far.^5^ Mutations of C or G nucleotides of Arg at positions 58 or 234 were most common (8/23). The clinical presentation of PNP deficiency is variable, but prognosis is poor, and without restoration of immune function most patients die from complications related to immunodeficiency.^1^–^3^ There have been no systematic studies of the genotype/phenotype correlation in PNP deficiency, but there is evidence of such correlation in its counterpart, ADA deficiency.^8^ The novel P146L missense mutation identified in patient 1 has some residual catalytic activity when expressed in *E coli.* By analogy with ADA deficiency, it is possible that this mutant allele might result in a somewhat milder clinical phenotype.

T-cell lymphopenia, recurrent infections, and neurologic findings should raise the suspicion of PNP deficiency, particularly if these are associated with low serum or urinary uric acid level (although the latter is not universally found).^9^ The diagnosis is usually established by measurement of PNP enzyme activity in red cells or blood mononuclear cells. Elevated serum or urinary levels of inosine and guanosine are confirmatory.^6^ Although B lymphocyte numbers have been normal in most cases, both of our patients had low B-cell numbers. However, patient 1 had high immunoglobulin levels, which may indicate B-cell dysregulation due to T-cell deficiency, rather than adequate B-cell function. Specific antibody titer to tetanus was normal in patient 2, which is not unusual to find in this disorder.^1^

Neurologically, both patients had motor developmental delay, a frequent feature of the disease. Various neurological abnormalities have been described including spastic diplegia or tetraplegia, ataxia, behavioral difficulties and mental retardation.^4^ Autoimmune phenomena occur in about one third of patients, mainly hemolytic anemia, thrombocytopenia and neutropenia. Patient 1 presented with relapsing neutropenia that was most probably autoimmune in nature; patient 2 had no evidence of autoimmunity.

In conclusion, this is the first description of Saudi patients with PNP deficiency, including one with a novel disease-causing mutation in the NP gene in one case. T lymphopenia, recurrent infections, and neurologic findings should raise the suspicion of PNP deficiency, especially with a history of parental consanguinity.
